# Risk Factors Associated with Mortality in *Acinetobacter baumannii* Infections: Results of a Prospective Cohort Study in a Tertiary Public Hospital in Guayaquil, Ecuador

**DOI:** 10.3390/antibiotics13030213

**Published:** 2024-02-23

**Authors:** Luz Abarca-Coloma, Miguel Puga-Tejada, Tamara Nuñez-Quezada, Otilia Gómez-Cruz, Carlos Mawyin-Muñoz, Shivan Barungi, Macarena Perán

**Affiliations:** 1 Critical Care Unit Hospital Teodoro Maldonado Carbo, Catholic University Santiago of Guayaquil, Guayaquil 090203, Ecuador; carlos.mawyin@cu.ucsg.edu.ec; 2 Instituto Ecuatoriano de Enfermedades Digestivas (IECED), Guayaquil 090505, Ecuador; mpuga@ieced.ec; 3 Department of Medical Microbiology Hospital Teodoro Maldonado Carbo, Catholic University Santiago of Guayaquil, Guayaquil 090203, Ecuador; tamara.nunez@cu.ucsg.edu.ec; 4 Infection Prevention and Control Program, Hospital Teodoro Maldonado Carbo, Guayaquil 090203, Ecuador; otilia.gomez@cu.ucsg.edu.ec; 5 Department of Health Sciences, University of Jaén, 23071 Jaén, Spain; sb000042@red.ujaen.es; 6 Excellence Research Unit “Modeling Nature” (MNat), University of Granada, 18010 Granada, Spain; 7 Biopathology and Regenerative Medicine Institute (IBIMER), University of Granada, 18010 Granada, Spain

**Keywords:** *Acinetobacter baumannii*, antimicrobial resistance, resistance, carbapenemase, colistin

## Abstract

Antibiotic overuse and the resulting antimicrobial resistance pose significant global public health challenges, providing an avenue for opportunistic pathogens like Acinetobacter baumannii to thrive. This study will report the trends of *Acinetobacter baumannii* antimicrobial resistance patterns at the Hospital Teodoro Maldonado Carbo, Ecuador. An observational, analytical, longitudinal, and prospective study was conducted involving patients diagnosed with hospital-acquired infections. Antimicrobial susceptibility testing was performed, followed by molecular analysis of carbapenemase genes in *Acinetobacter baumannii* isolates. We included 180 patients aged from 16 to 93 years. The hospital mortality rate was 63/180 (35%). Invasive mechanical ventilation (IMV) was indicated in 91/180 patients (50.4%). The overall survival (OS) rate in patients on IMV was 49.5% (45/91), with a median survival of 65 days. The OS rate in patients not on IMV was 80.9% (72/89), with a median survival of 106 days (HR 2.094; 95% CI 1.174–3.737; *p* = 0.012). From multivariate analysis, we conclude that ventilator-associated pneumonia is the most related factor to OS.

## 1. Introduction

*Acinetobacter baumannii* is a gram-negative coccobacillus that can be found in the environment, soil, plants, inanimate surfaces, and even on the skin. *Acinetobacter baumannii* has great clinical importance as it can infect patients with chronic diseases and comorbidities, as well as those that have undergone surgery and other invasive procedures such as vascular and urinary catheters, parenteral nutrition, tracheostomy, or invasive mechanical ventilation (IMV). Such patients might develop several medical conditions like bacteremia, sepsis, ventilator-associated pneumonia (VAP), postsurgical meningitis, urinary tract infections, surgical wound infections, etc. Therefore, it is necessary to administer a wide range of antibiotics to which *Acinetobacter baumannii* could be resistant. Multiple drug resistance (MDR) is defined as a bacterium with non-susceptibility to at least one agent in three antimicrobial categories. Extensive drug resistance (XDR) indicates non-susceptibility to at least one agent in almost all the antimicrobial categories. Pan-drug resistance (PDR) is non-susceptibility to all the antimicrobial categories [1,2].

Carbapenem-resistant *Acinetobacter baumannii* (CRAB) and extended CRAB (XCRAB) have been defined as the resistance of *Acinetobacter baumannii* to β-lactams, aminoglycosides, fluoroquinolones, carbapenems, and every drug except for tigecycline and colistin [1,3]. *Acinetobacter baumannii’s* resistance to carbapenems has led to high mortality due to hospital-acquired infections, and its increasing drug resistance calls for the development of new antibiotics [4]. This pathogen has become very interesting over the last decade due to its natural MDR phenotype, which acquires new resistance mechanisms [5]. *Acinetobacter baumannii* is considered an opportunistic bacterium associated with high morbidity and mortality, being a common Intensive Care Unit (ICU) guest [6,7].

Acinetobacter is often transmitted to patients through exposure to an infected environment and through contaminated hands of health workers. The predisposition to these infections is caused by the pressure of colonization, extended-spectrum antibiotic exposure selection, and disruption of anatomic barriers (for example, catheter placement or endotracheal tubes, and traumatic or surgical skin and integument lesions). Infections with *Acinetobacter baumannii* are associated with IMV, intravenous and urinary catheterization, surgery, invasive procedures, and extended-spectrum antimicrobial procedures, especially in patients presenting burns, traumatology patients, and those in the ICU [8]. This nosocomial pathogen can live for long periods on different surfaces due to the development of biofilms [9]. The main infections include (i) IMV exposure and consequent VAP; (ii) infections associated with intravascular devices; (iii) surgical wound infections; (iv) bloodstream infections; (v) urinary tract infections; and (vi) postsurgical meningitis [10]. Although it is mainly a pathogen associated with medical care, *Acinetobacter baumannii* infections can also occur outside medical care facilities, as reported in wounded soldiers of the Gulf War [11] and in the victims of the Turkey earthquake [12,13]. Furthermore, *Acinetobacter baumannii* has been spotted in tropical and subtropical regions as a community-acquired pathogen associated with relatively high mortality [8].

Often, this organism invades patients with comorbidities, antibiotic exposure, and recent hospitalization, which makes it complicated to determine whether it is the pathogen causing an infection of interest, particularly in patients with non-sterile localized infections such as pneumonia and wound infection [14]. Infections caused by *Acinetobacter baumannii* have been identified globally and are increasing. It is the cause of 2–10% of all gram-negative infections in the ICU of the USA and Europe [15]. The resistance of *Acinetobacter baumannii* to antibiotics is due to a combination of mechanisms that include a waterproof cell membrane, an increase in outflow pumps, extended spectrum β-Lactamases (ESBL), Metallo-β-Lactamase (MBL), and carbapenem-hydrolyzing class D β-Lactamase (CHDL) [16,17].

In Latin America, several cases of *Acinetobacter baumannii* have been reported. The situation with *Acinetobacter baumannii* CRAB/XCRAB is especially worrying in developing countries like Ecuador, where hospital conditions and policies are not updated, increasing the nosocomial infection risk [18]. Here, we describe the healthcare-associated infections caused by *Acinetobacter baumannii* that were identified in different units at the Hospital Teodoro Maldonado Carbo (HTMC), Guayaquil, Ecuador. The clinical and molecular epidemiology of carbapenem-resistant *Acinetobacter baumannii* is analyzed together with patient characteristics such as comorbidities, gender, age, and surgeries in order to identify the susceptibility profiles of patients to promote a more effective action protocol.

## 2. Results

A total of one hundred eighty (180) patients with Acinetobacter baumannii infections were included in this study. Signed informed consent for research purposes was obtained from all patients with Acinetobacter baumannii infection or their legal guardians. Patients and hospital areas presenting such cases and their numbers are summarized in Table 1. Four units, i.e., the Senior Observation Unit, Intensive Care Unit, and Traumatology and Neurological Care Unit, were responsible for 129 infected patients, that is, 71% of the patients included in this study.

The demographic characteristics of the study population are summarized in Table 2, including 58% women and 42% men with *Acinetobacter baumannii* infections, identified along a median of 31.5 days of hospitalization. Overall, 63 fatalities (35% mortality rate) were observed in patients with *Acinetobacter baumannii* infections (Table 3). The causes of hospitalization of infected patients are described in Table 3. The most predominant causes were cerebrovascular accident (32.2%), limb trauma (13.9%), and chronic renal failure (9.4%).

Clinical characteristics related to the different types of infections are summarized in Table 4. Pneumonia represented 49.9% of infected patients, 13.9% of patients had urinary tract infections, 10% had surgical wound infections, 9.4% had bacteremia not related to other sites of infection, 3.3% suffered from osteoarticular infections, and 1.7% had surgical meningitis. Regarding associated risk factors, 91/180 patients (50.4%) had IMV-associated infections.

An extensive statistical analysis relating different factors with mortality due to hospital-acquired infection is summarized in Table 5 and Table 6. Univariate Cox regression concluded that the main risk factors associated with high mortality were a history of chronic renal failure (*p* < 0.001), hemodialysis (*p* < 0.001), piperacillin plus tazobactam resistance (*p* = 0.004), a history of high blood pressure (*p* = 0.003), IMV exposure (*p* = 0.005), imipenem resistance (*p* = 0.022), and meropenem resistance (*p* = 0.029) (Table 5). A multivariate analysis (Table 6) revealed that IMV exposure was the most important factor associated with the patients’ mortality (*p* = 0.012), followed by hemodialysis (*p* = 0.022) and a history of high blood pressure (*p* = 0.041). It is important to highlight the potential mutually excluded association between hemodialysis and a history of high blood pressure: 20/80 patients with a history of high blood pressure were under hemodialysis (25%), but 20/27 patients under hemodialysis had a history of high blood pressure (74%).

**Figure 1:**
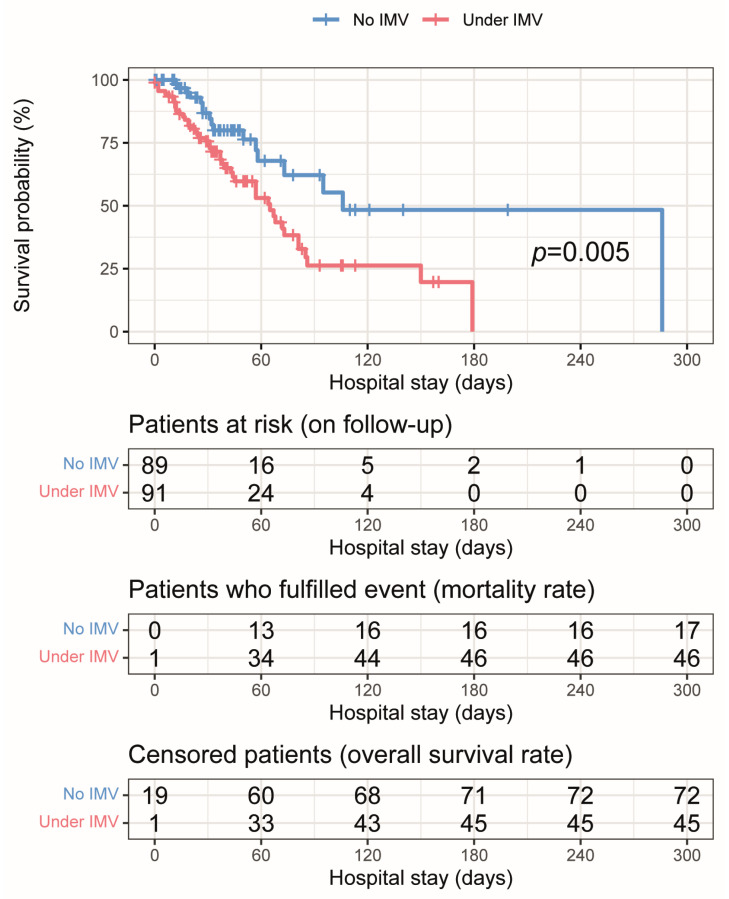
Kaplan–Meier curve of the survival probability over hospital stay (days) associated with invasive mechanical ventilation (IMV) in patients with Acinetobacter baumannii infection.

Figure 1 shows the overall survival (OS) associated with invasive mechanical ventilation (IMV) in patients with *Acinetobacter baumannii* infection. The OS rate in patients on IMV (red curve) was 49.5% (45/91), with a median survival of 65 days (95% CI 1.90–2.70). The OS rate in patients not on IMV (blue curve) was 80.9% (72/89), with a median survival of 106 days (IC 95% 2.43–6.00). A 2× risk of death was seen in patients on IMV (HR 2.203; IC 95% 1.244–3.900; *p* = 0.005).

Finally, the genetic characteristics of the isolated bacteria and their antibiotic resistance were analyzed (Table 7). A total of 85 (47.22%) corresponded to Acinetobacter baumannii MDR; 42 (23.33%) corresponded to XDR, and 1 (0.5%) corresponded to PDR. Acinetobacter baumannii resistance was recorded as follows: 54% resistant to ampicillin plus sulbactam, 87% resistant to ceftazidime, 72% resistant to ceftriaxone, 80% resistant to ciprofloxacin, 82% resistant to imipenem, 75.6% resistant to meropenem, and 5% resistant to tigecycline. In addition, 87.7% of the patients were given antibiotic therapy as follows: 45 (25%) were given meropenem, 8 (4.4%) were given imipenem, and 62 patients (34.4%) were transferred to other healthcare centers.

Regarding treatment, combined therapy was used: ampicillin plus sulbactam and colistin: 22 (12.7%); colistin plus meropenem: 45 (25%); colistin plus tigecycline: 6 (3.3%); meropenem plus tigecycline: 3 (1.6%); doxycycline plus fosfomycin: 1 (0.5%); triple scheme with colistin–doxycycline–meropenem: 19 (10.5%); meropenem plus ampicillin plus sulbactam plus colistin: 7 (3.8%); and colistin–tigecycline–meropenem: 3 (1.6%). Monotherapy with colistin, ampicillin plus sulbactam, meropenem, tigecycline, and doxycycline was also administered (Table 8).

## 3. Discussion

*Acinetobacter baumannii* is an opportunist pathogen responsible for large infection outbreaks worldwide [19,20,21]. Pneumonia associated with *Acinetobacter baumannii* infection, as we have shown here, has been described before in other studies. In our study, the mortality rate of infected patients was 35%. This was similar to the study at the Manuel Gea González hospital in Mexico, in which 33.3% of deaths were associated with *Acinetobacter baumannii* [22]. Furthermore, in a study carried out in China, 71.2% of ICU patients presented with Acinetobacter baumannii infection, and 86.5% XDR of these cases suffered from hospital-acquired pneumonia [23]. Another study carried out in Porto Alegre, Brazil, at the Nossa Senhora da Conceicao Hospital with 153 patients under IMV showed that 45% of those patients presented pneumonia associated with IMV with the presence of *Acinetobacter baumannii* [24].

In our study, the thirty-day mortality rate of infected patients was 35%, similar to the study at the Manuel Gea González hospital in Mexico, with 33.3% of global mortality associated with *Acinetobacter baumannii* [22]. In the study carried out at a hospital in China, the mortality rate was 42.8% [20]. Furthermore, the mortality rate found at the ICU at the Nossa Senhora da Conceicao in Porto Alegre, Brazil, was 66% [24].

Regarding antimicrobial susceptibility, our results were slightly higher than antibiotic resistance patterns found in other studies carried out in hospitals worldwide. For instance, in a study performed in two medical centers in Guayaquil City, 33 out of 35 isolates of Acinetobacter baumannii presented resistance to all β-lactam antibiotics (94.2%) [25], higher than the 75.6% demonstrated in the present study. Surveillance studies in Brooklyn, NY, including 1286 *Acinetobacter baumannii* patient isolates from 15 hospitals, showed that 30% were resistant to three or more types of antibiotics (carbapenems, fluoroquinolones, and aminoglycosides) [26,27]. A multicenter study was carried out from June 2017 to June 2018 in 12 tertiary healthcare centers in Italy, including all hospitalized patients with MDR *Acinetobacter baumannii* bacteremia. A total of 281 cases were detected, and 98 cases (34.8%) were classified as primary bacteremia. Infected patients were isolated from the following hospital units: 83% of the patients were from the ICU, 9.7% from the general medical unit, 3.6% from the surgical unit, and 1.7% patients from the emergency unit. Those patients presented resistance to the following antibiotics: colistin, 1.4%; gentamicin, 87.3%; amikacin, 89.1%; and meropenem, 100%. Based on the information above, 98.6% of *Acinetobacter baumannii* strains were XDR and 1.4% were considered PDR [28]. Comparing the information in this study, these were the results: outpatient service: 8.3%; hospitalization: 36.7%, critical area: 55%. It was observed that 47.22% presented *Acinetobacter baumannii* MDR infections, 23.33% presented *Acinetobacter baumannii* XDR infections, and 1 case (0.5%) corresponded to *Acinetobacter baumannii* PDR infections. Of all these patients, 9.4% had bacteremia.

A study carried out at a medical complex in Durban, South Africa, from January 2008 to December 2014 showed that, in the sepsis group, *Acinetobacter baumannii* was resistant to multiple drugs (MDR) in 53–60%, extremely resistant (XDR) in 1–19%, and pan-resistant (PDR) in 1% [29].

In a prospective multicenter study carried out in Turkey for 6 months on the evaluation of antimicrobial resistance to multiple drugs in *Acinetobacter baumannii* infections, resistance rank results were as follows: amikacin, 91.8%; ampicillin/sulbactam, 99.4%; ceftazidime, 99.4%; ciprofloxacin, 100%; imipenem, 99.4%; colistin, 1.2%; and tigecycline, 1.7% [30].

Acinetobacter can develop several mechanisms to resist antibiotic treatment, which results in strains that are resistant to every antibiotic [31]. It also leads to an increasing trend in MDR strains; XDR strains; and, on a lower level, PDR strains, which restricts treatment options. The production of carbapenems is among the resistant mechanisms, and they can be classified as follows: type A: serine carbapenem (KPC, IMI); type B: MBL (IMP, VIM, SIM, and NDM-1); or type D: oxacillin (OXA), the last one being the most common in the world [21,32]. Molecular biology tests were negative in the search for intrinsic β-lactamase genes. The production of other types of oxacillin that justify resistance, such as OXA 24/40 or OXA 72, was not disregarded [25]. In the present study, resistance in the isolations was observed to imipenem and meropenem. Therefore, carbapenem resistance can be the result of previous enzymatic mechanisms not assessed in this study.

In our study, the risk factors associated with high mortality agree with those found in the study of Djordjevic [33]. It was evident that the mortality risk factors in patients with hospital-acquired infections caused by CRAB were exposure to IMV, having been treated in other hospital units, and having been medicated with carbapenems. At the public hospital of Turkey, the risk factors of *Acinetobacter baumannii* resistance to infection with carbapenems found in the analysis showed that the use of IMV had a rank of *p* = 0.016; stays shorter than 15 days in the ICU had a rank of *p* < 0.001; and a history of using carbapenems had a rank of *p* < 0.001, among other independent risk factors [34]. The multicenter study carried out in Turkey showed that long-term supply of drugs at the ICU, bacteremia associated with ventilation, use of third-generation cephalosporins before the diagnosis of an infection, and liver cirrhosis were important risk factors in the mortality of patients infected with *Acinetobacter baumannii* [30]. According to the cited studies, due to some characteristics, such as tolerance to desiccation, *Acinetobacter baumannii* has become a successful opportunistic pathogen in the nosocomial environment [35]. Its ability to become attached to glass coverslips and to form biofilms greatly increases its survival chances in dry conditions compared to strains that do not form biofilms [36]. This characteristic allows them to stick to medical devices in patients in the ICU, where high frequencies of *Acinetobacter baumannii* infections can be detected.

One of the most common invasive procedures carried out in hospital intensive care units is intubation. This procedure eliminates physiological functions such as warming, humidification, and purification of the superior respiratory membrane. Therefore, the risk of developing pneumonia associated with IMV assistance is increased [37]. Biofilm formation in the endotracheal tract and micro suction of oropharyngeal secretions contaminated by endogenous flora moves pathogens towards the distal respiratory tract, while the removal of pathogens from the trachea is reduced due to ciliary tracheal low movement and deteriorated cough [36]. These are relevant mechanisms that cause the development of VAP. Among the prevention methods for pneumonia associated with IMV, we have the following categories: interruption of sedation, early weaning, high level of the bed headboard, and oral health care. The chosen Intensive Care Units have added new endotracheal tubes covered with silver sulfate or with modified sleeves to avoid aspiration and secretion drainage through continuous or intermittent subglottic vias. There are also new devices to eliminate the biofilm from the endotracheal tube, to apply saline solution before suction, and to perform early tracheostomy [38,39].

Regarding antibiotic treatment, the current tendency is the use of a combination of two or three therapies that include colistin or tigecycline. Some schemes use high doses of tigecycline, like 100 mg every 12 h and standard doses of 50 mg IV every 12 h. For meropenem, a 2 gm dose every 8 h is used as a standard dose, and a loading dose is used for colistin [22]. Antibiotics used at the Nossa Senhora da Conceicao from Porto Alegre, Brazil, were the following: polymyxin B plus meropenem, polymyxin B plus ceftazidime, polymyxin B plus ampicillin–sulbactam, polymyxin B plus amikacin, ampicillin–sulbactam plus polymyxin B plus tigecycline, and ceftazidime plus amikacin. Ampicillin sulbactam was also used in a 3 gm IV dose every 4 h and a dose of amikacin of 20 mg per kg of weight per day [24]. In the study described herein, of the 128 patients presenting resistance, 122 received proper treatment, and 6 patients did not receive the intended treatment for *Acinetobacter baumannii* due to a late diagnosis. The combined therapy included colistin plus meropenem, ampicillin plus sulbactam, or tigecycline, like in the double therapy. This is in agreement with other studies that have suggested tigecycline as a therapy option against *Acinetobacter baumannii* [7]. In cases of triple schemes, colistin and meropenem plus ampicillin sulbactam were used.

## 4. Materials and Methods

- Experimental setting and study population

This observational, analytic, prospective, longitudinal cohort-type study was performed in outpatient service and hospitalized patients at the HTMC between January 2017 and December 2018. Patients diagnosed with hospital-acquired infections (HAI) who had been isolated due to *Acinetobacter baumannii* infection and colonization were included in this study. The research protocol was approved by the Research Department of the HTMC and by the Ethics Committee (HLV-DOF-CEISH-027). Patients or their legal guardians were required to sign an informed consent form before being included in this study.

On the premises, data were gathered from the Medical Record Registration System of the AS-400 of the patients who had been isolated due to *Acinetobacter baumannii* infection. Information from all patients was collected, and a database was created including age, gender, area to which the person belonged, prior hospital admissions, comorbidities, use of central and vesical catheters, surgeries, prior antibiotic therapy, sample collection location, type of infection, and treatment given to the patient.

- Collection of samples

Clinical isolates for *Acinetobacter baumannii* were collected from sputum samples, tracheal aspirates, wounds, catheters, and urine. Blood sample cultures and respiratory samples were grown in MacConkey, blood, and chocolate agar. The process of isolating and identifying *Acinetobacter baumannii* was performed utilizing microbiological techniques described in the standardized proceeding manuals.

*Acinetobacter baumannii* MDR strain confirmation was performed through the automated Vitek 2 compact (BIOUMERIEUX) system for the identification of isolates and tests of susceptibility to antimicrobial agents, as established by the Clinical and Laboratory Standards Institute guidelines (CLSI). The following antibiotics were tested: amikacin, ampicillin/sulbactam, cefepime, cefoxitin, ceftazidime, ceftriaxone, ciprofloxacin, colistin, doripenem, ertapenem, extended-spectrum beta-lactamases (ESBL), gentamicin, imipenem, meropenem, piperacillin/tazobactam, and tigecycline. Isolated strains were classified as resistant to more than three drugs, or multidrug-resistant (MDR); resistant to all drugs but one or two, or extensively drug-resistant (XDR); and resistant to all drugs, or pan-drug-resistant (PDR).

- Statistical analysis

*Technical considerations*. A *p*-value < 0.05 was statistically significant. Data were analyzed in R v4.0 (R Foundation for Statistical Computing; Vienna, Austria).

*Descriptive statistics*. Numeric variables were described by mean (standard deviation, SD) or median (interquartile range, IQR), in agreement with the statistical distribution (Kolmógorov–Smirnov test). Categorical variables were described by percentages, with the corresponding 95% confidence interval (CI) when appropriate.

*Inferential statistics*. There were study variables established as potential mortality risk factors through Cox regression (Hazard Ratio, HR), considering hospital stay (days) as time and mortality as the event. Following a stepwise approach, study variables with significant univariate associations with the event continued to multivariate Cox regression. In the case of mutually exclusive study variables in clinical terms, the authors decided by consensus which study variable must not continue to the multivariate Cox regression.

## 5. Conclusions

The most frequent type of infection associated with healthcare for *Acinetobacter baumannii* at the HTMC was pneumonia. The most important factor associated with mortality was IMV and the consequent VAP, followed by hemodialysis and history of chronic renal failure. Early diagnosis in patients with risk factors in critical areas and prompt treatment for *Acinetobacter baumannii* infections could decrease mortality.

The high rates of resistance to carbapenems recorded in all the studies performed in MDR, XDR, and PDR strains urge us to use other therapeutic options, such as colistin and tigecycline, which show lower resistance. These factors also recommend discontinuing the use of carbapenems and using antibiograms for specific treatment, as well as personalizing the treatment for each patient.

Novel protocols should be applied in patients under IMV or hemodialysis, such as devices to avoid biofilms from the endotracheal tube, secretion drainage through continuous or intermittent subglottic vias, application of saline solution before suction, and early tracheostomy.

## Figures and Tables

**Table 1:** Infection frequency according to hospital units.

Hospital Unit	Number of Patients Infected	Frequency Related to the Number of Patients in Each Hospital Unit
Neurological Care Unit	18	2.65%
Endocrinology Hospitalization	1	0.15%
Intensive Care Unit	39	5.74%
Neurology Unit	2	0.29%
Traumatology Hospitalization	18	2.65%
Senior Observation	54	7.95%
Infectiology Hospitalization	3	0.44%
Internal Medicine Unit	6	0.88%
Pulmonology Unit	10	1.47%
Emergency Unit	5	0.73%
Nephrology Unit	6	0.88%
Ophthalmology Hospitalization	1	0.14%
Urology Outpatient Service	6	0.88%
Gynecology Outpatient Service	3	0.44%
High Obstetrical Risk	4	0.59%
Plastic Surgery Hospitalization	3	0.44%
Hematology Unit	1	0.14%

**Table 2:** Demographic characteristics of the studied population.

	(*n* = 180)
**Age (years old, yo)**, median (IQR)	57.5 (38–71.3)
Pediatric (<18 yo)	7 (3.9)
Young adults (18–39 yo)	39 (21.7)
Adults (40–64 yo)	70 (38.9)
Elderly (≥65 yo)	64 (35.6)
**Gender (female)**, *n* (%)	
Female	58 (32.2)
Male	122 (67.8)
**History of diabetes**, *n* (%)	47 (26.1)
**History of high blood pressure**, *n* (%)	81 (45.0)

**IQR**, interquartile range.

**Table 3:** Clinical characteristics of patients included in the study.

	(*n* = 180)
**Hospital lethality**, *n* (%)	63 (35.0)
**Hospital department**, *n* (%)	
Outpatient service	15 (8.3)
Hospitalization	66 (36.7)
Critical area	99 (55.0)
**Underlying disease**, *n* (%)	
Cerebrovascular accident	58 (32.2)
Limb trauma	25 (13.9)
Chronic renal failure	17 (9.4)
Pneumonia	16 (8.9)
Acute surgical abdomen	12 (6.7)
Cancer	11 (6.1)
Skin and subcutaneous tissue infections	10 (5.6)
Urinary tract infection	9 (5.0)
Preeclampsia, eclampsia, or HELLP syndrome	8 (4.4)
Rheumatoid arthritis	6 (3.3)

**Table 4:** Infection under study: type, study sample, and possible associated factors.

	(*n* = 180)
**Type of infection**, *n* (%)	
Pneumonia	88 (48.9)
Urinary tract infection	25 (13.9)
Surgical wound infection	18 (10.0)
Bacteremia not related to other sites of infection	17 (9.4)
Skin and soft tissue infection	13 (7.2)
Upper respiratory tract infection	10 (5.6)
Osteoarticular infection	6 (3.3)
Postsurgical meningitis	3 (1.7)
**Anatomical sites of isolation**, *n* (%)	
Tracheal aspirate	71 (39.4)
Urine culture	25 (13.9)
Surgical wound	18 (10.0)
Sputum	17 (9.4)
Tissue sample	12 (6.6)
Bronchoalveolar lavage	10 (5.5)
Blood culture	9 (5.0)
Catheter culture	8 (4.4)
Rectal swab	6 (3.3)
Cerebrospinal fluid	3 (1.7)
Pleural fluid	1 (1.1)
**Associated risk factors**, *n* (%)	
Invasive mechanical ventilation (IMV)	91 (50.6)
Other invasive techniques	157 (87.2)

**Table 5:** Relationship between different variables under study vs. death related to the infection under study during hospital stay: univariate Cox regression.

Variable	HR (95% CI; *p*-Value)
Gender (female)	1.694 (1.003–2.883; 0.050)
Prior hospitalization in the last 90 days	0.985 (0.590–1.643, 0.950)
History of diabetes	1.602 (0.950–2.715; 0.079)
History of high blood pressure	2.140 (1.281–3.550; 0.003)
History of chronic renal failure	4.114 (2.133–7.891; <0.001)
Nosocomial infection	0.612 (0.245–1.561; 0.300)
Invasive mechanical ventilation (IMV) exposure	2.203 (1.244–3.900; 0.005)
Time spent under mechanical invasive ventilation	1.003 (0.989–1.018; 0.620)
Invasive techniques practice	0.598 (0.215–1.673; 0.310)
Central venous catheter placement	2.238 (0. 958–5.199; 0.062)
Hemodialysis	3.090 (1.617–5.859; <0.001)
Time spent in the Intensive Care Unit	0.994 (0.985–1.005; 0.299)
Ceftriaxone resistance	0.474 (0.113–1.992; 0.310)
Imipenem resistance	0.433 (0.215–0.894; 0.022)
Meropenem resistance	0.439 (0.209–0.920; 0.029)
Piperacillin plus tazobactam resistance	0.477 (0.288–0.795; 0.004)
Tigecycline resistance	0.773 (0.460–1.324; 0.340)

**CI**, confidence interval; **HR**, hazard ratio.

**Table 6:** Relationship between different variables under study vs. death related to the infection under study during hospital stay: multivariate Cox regression.

Variable	HR (95% CI; *p*-Value)
History of high blood pressure	1.732 (1.022–2.933; 0.041)
Invasive mechanical ventilation exposure	2.094 (1.174–3.737; 0.012)
Hemodialysis	2.201 (1.119–4.330; 0.022)
Imipenem or meropenem resistance	0.593 (0.239–1.468; 0.258)
Piperacillin resistance	0.594 (0.347–1.015; 0.056)

**CI**, confidence interval; **HR**, hazard ratio.

**Table 7:** Resistance phenotypes of the isolated bacteria in infected patients.

	(*n* = 180)
**Phenotype pattern**, *n* (%)	
MDR	85 (47.22)
PDR	1 (0.5)
XDR	42 (23.33)
**Antibiotic resistance**, *n* (%)	
Ampicillin plus sulbactam	98 (54)
Meropenem	136 (75.6)
Colistin	3 (2.5)
Tigecycline	9 (5)

**Table 8:** Antibiotic schemes.

Frequency of Prescription	(*n* = 180)
**Monotherapy**, *n* (%)	
Meropenem	81 (45.0)
Colistin	67 (37.2)
Tigecycline	13 (7.2)
**Double scheme**, *n* (%)	
Colistin + meropenem	45 (25)
Ampicillin sulbactam + colistin	22 (12.5)
Colistin + tigecycline	6 (3.3)
**Triple scheme**, *n* (%)	
Colistin + doxycycline + meropenem	19 (10.5)
Meropenem + ampicillin sulbactam + colistin	7 (3.8)
Colistin + tigecycline + meropenem	3 (1.6)

## Data Availability

Data are unavailable due to privacy or ethical restrictions.
